# Severe Acro-osteolysis in a Case of Limited Cutaneous Scleroderma

**Published:** 2017-01

**Authors:** Milad HOSSEINIALHASHEMI, Babak DANESHFARD, Omid KESHAVARZIAN

**Affiliations:** 1. Student Research Committee, Shiraz University of Medical Sciences, Shiraz, Iran; 2. Research Center of Quran, Hadith and Medicine, Shiraz University of Medical Sciences, Shiraz, Iran; 3. Essence of Parsiyan Wisdom Institute, Phytopharmaceutical Technology and Traditional Medicine Incubator, Shiraz University of Medical Sciences, Shiraz, Iran

## Dear Editor-in-Chief

Systemic sclerosis (scleroderma) is a rare fetal autoimmune disease of connective tissue leading to skin fibrosis as well as internal organ involvement (1). An annual incidence rate of 2–20 and prevalence rate of 100–300 per million population have been estimated for scleroderma with a mean survival of 12 yr from diagnosis (2–4). Moreover, there is a considerable preponderance of female gender and black race among scleroderma patients (5). The disease is divided into diffuse and limited cutaneous subtypes. Despite diffuse form, skin involvement in limited cutaneous subtype of scleroderma is limited to face, forearm, hands, and feet (2, 6). This type of scleroderma is accompanied with CREST syndrome, which includes “Calcinosis, Raynaud phenomenon, Esophageal involvement, Sclerodactyly, and Telangiectasias” (2, 7). Acro-osteolysis is another clinical feature of the disease which is the result of bone resorption in terminal phalanges (8). Its identification is a confirmative criterion in diagnosis of scleroderma (9).

**Fig. 1:**
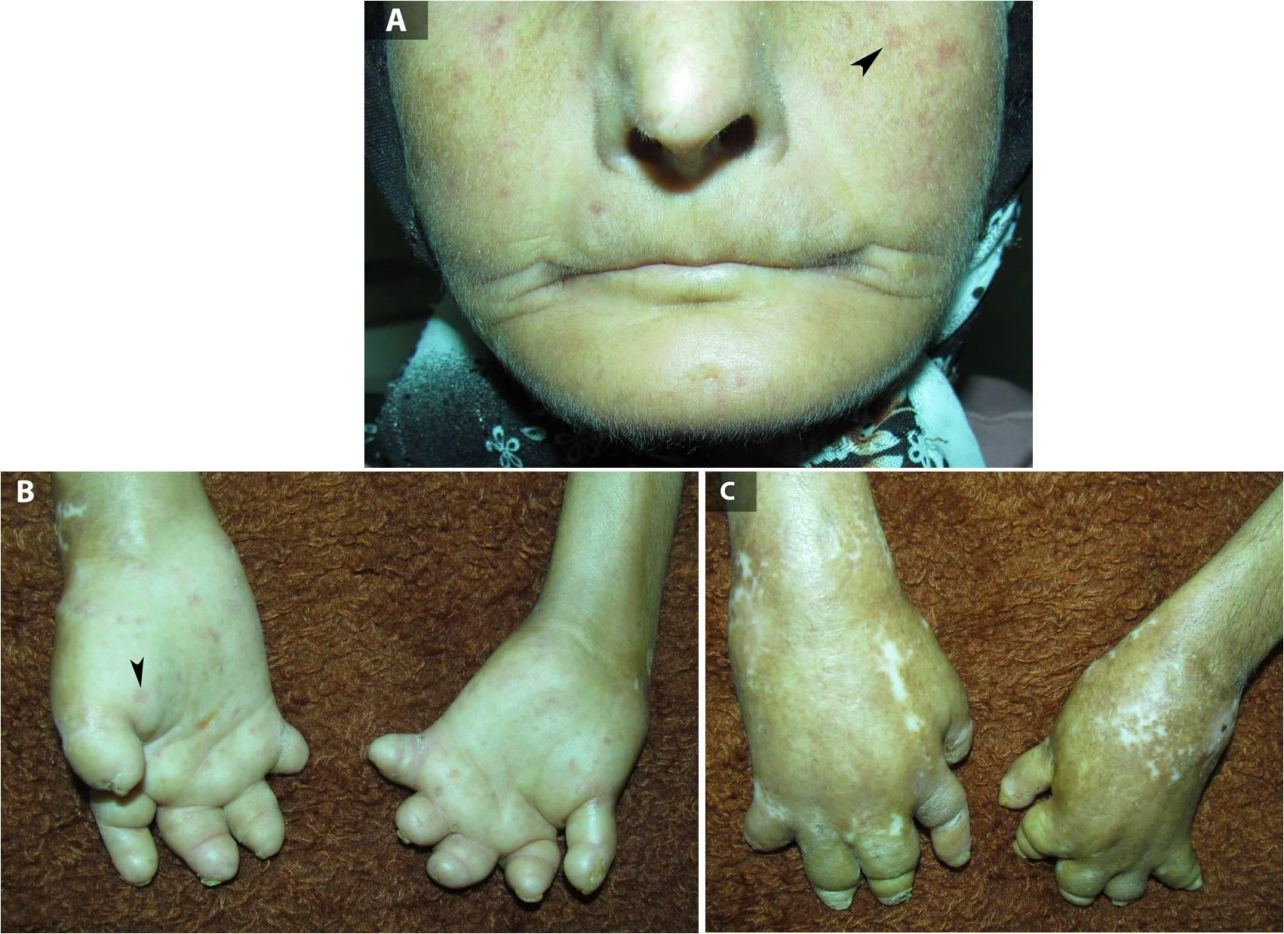
Pursed lips (A), cutaneous telangiectasia (A and B, arrowheads), acro-osteolysis (B and C), and salt-and-pepper appearance (C) in a 43-year-old woman with limited cutaneous scleroderma.

A 43-year-old woman with a 30-yr history of limited cutaneous scleroderma presented with an infected left foot ulcer of 3-weeks duration. She had a positive history for Raynaud phenomenon and digital ulcers. The patient had no fatigue, muscle weakness, pain, cardiopulmonary and/or gastrointestinal problems. Physical examinations revealed skin sclerotic involvement in face and extremities, cutaneous telangiectasia, pursed lips, shortened digits (acro-osteolysis), regions of skin hypo- and hyperpigmentation (salt-and-pepper appearance), and calcinosis cutis at the elbows and forearms (Fig. 1). At the end, her left lower limb was amputated below the knee. She recovered after the operation uneventfully.

Such a severe form of acro-osteolysis in limited cutaneous scleroderma is rarely seen because of uncontrolled disease. Early diagnosis is crucial to improve the treatment outcome (10).
